# A Scientometric Analysis of the 50 Most Cited Articles for Reconstruction of the Lower Extremity

**DOI:** 10.1155/2019/3068028

**Published:** 2019-01-28

**Authors:** Zacharia Mbaidjol, Jens Rothenberger, Rajesh Chetany

**Affiliations:** ^1^Plastic and Reconstructive Surgery, Queen Victoria Hospital, East Grinstead, West Sussex RH19 3DZ, UK; ^2^BG Unfallklink an der Universität Tübingen, Klinik für Hand, Plastische, Rekonstruktive und Verbrennungschirurgie, Tübingen, Germany; ^3^FMI Spital Interlaken, Weissenaustrasse 27, 3800 Interlaken, Switzerland

## Abstract

**Background:**

Lower extremity reconstruction has always been a challenge. Some of the published articles had a major impact on the field but are often not considered as classics because they have fewer citations. We therefore conducted a scientometric analysis of the most cited articles with a focus solely on the lower limb.

**Methods:**

A search was conducted on Medline, the Web of Science database, Google Scholar, and Scopus identifying articles relevant to reconstructive surgery of the lower limb. All journals were included with no time frames. Articles relating solely to orthopedics or vascular reconstruction were excluded. The number of citations obtained were then plotted and compared between the different search engines. The mean citation number was calculated by taking into consideration the total number of years since the article's first year of publication. Articles were then ranked and classified according to their authors, their years of publications, and their countries. They were furthermore categorized and analyzed.

**Results:**

Highly cited articles were easily retrieved with Google Scholar, mostly published in Plastic and Reconstructive Surgery (*n* = 37) and were mainly authored by American Medical Centers (*n* = 22). Fifty-four percent (54%) of these classic articles discussed the design of new flaps or were anatomical studies.

**Conclusions:**

We were not able to find a correlation between the year of citation and the number of citations. The citation pattern of a paper cannot be predicted, but a majority of highly cited article allowed the design of new reconstructive techniques.

## 1. Introduction

There are over 24000 peer-reviewed journals [1] and more than 237 medical disciplines. To find articles that have high relative importance to the research topic is a daunting task. Garfield in response to this challenge devised the impact factor in 1960 [2]. It was intended to serve as a way to help scientists browse with more ease and effectiveness through this magnitude of journals by identifying articles most often cited and by proxy more relevant.

The impact of an article is mostly observed the following 2 years after its publication. The impact factor of a journal is defined as the number of citations a journal has received in that current year to the number of published articles within the two previous years, divided by the number of articles published by that journal in the same two years [2]. Journals with the highest impact factors are associated with quality and prestige [3], and the articles they publish tend to be more cited. The impact factor therefore remains high in these journals because of the well-known 80/20 rule which results in 20% of the articles accounting for 80% of the citations [2]. Breakthrough articles can have over 300.000 articles. This lead to the term “Citations classic articles” introduced in 1977 by Eugene Garfield, selecting some of the most cited papers ever published in science.

In the field of reconstructive surgery, all previously written citation articles limited their search criteria to main plastic surgery journals and either encompassed the whole field of plastic surgery or a specific branch [4–7].

Which are the 50 most cited articles in regard to reconstruction of the lower limb? The aim of these articles is to acknowledge the most cited articles referring to lower extremity reconstruction because of the challenging nature of these procedures. This article gives an overview of how most procedures and techniques now in place came into existence and the different techniques that came to be before being replaced by another in order to overcome the limitations of the previous technique.

## 2. Methods

A search was conducted on Medline, the Web of Science database (WoS), Google Scholar (GS), and Scopus identifying articles relevant to reconstructive surgery of the lower limb. All journals were included with no time frame. We used specific thesaurus (Mesh Terms) terms and keywords as follows: “lower extremity,” “lower limb,” “legs,” “reconstruction,” “flaps,” “plastic surgery,” and “microsurgery.” On GS, an advanced search was performed with a combination of our keywords as exact phrases. We excluded in a first step duplicate articles among the databases, using EndNote (Endnote X8.2, Clarivate analytics, Philadelphia, PA, USA). In a second step, we went through all the titles and abstracts. All articles that did not include our important terms and keywords on the abstract and titles were excluded. We also did not consider those focusing on the lower extremity as a donor site for reconstruction of other parts of the body and those relating solely to orthopedics or vascular reconstruction. The search result of each database was also used to retrieve articles in the other databases as a mean to evaluate consistency of the results.

In the final step, we noted the years with the most citations, the publishing journal, and the country of publication for each article and the mean citation number was calculated by dividing the number of citations by the number of years since the article's first year of publication. We furthermore compared the differences in citation ranking for the 10 most cited articles according to the three abovementioned main search engines. These top 10 articles were then further evaluated by plotting the number of citations per year according to each year since their initial publication to see if a citation pattern existed. Three authors extracted the data from the full-articles examined. Discrepancies between two reviewers were resolved by discussion. A third author performed an independent search, and results were compared to verify accuracy.

## 3. Results

The most cited article entitled “The free vascularized bone graft: a clinical extension of microvascular techniques [8]” was published in plastic and reconstructive surgery (PRS) in 1975 and has been cited 829 times according to the WoS database. The 50^th^ most cited article “The instep of the foot as a fasciocutaneous island and as a free flap for heel defects” was referenced 106 times also according to the WoS (Table 1). These 2 articles were both published in PRS 6 years apart. Taylor is the most cited author with three (3) articles listing as the first author.  Table 2 shows a list of 6 other authors with multiple entries (Table 2).

**Figure 1:**
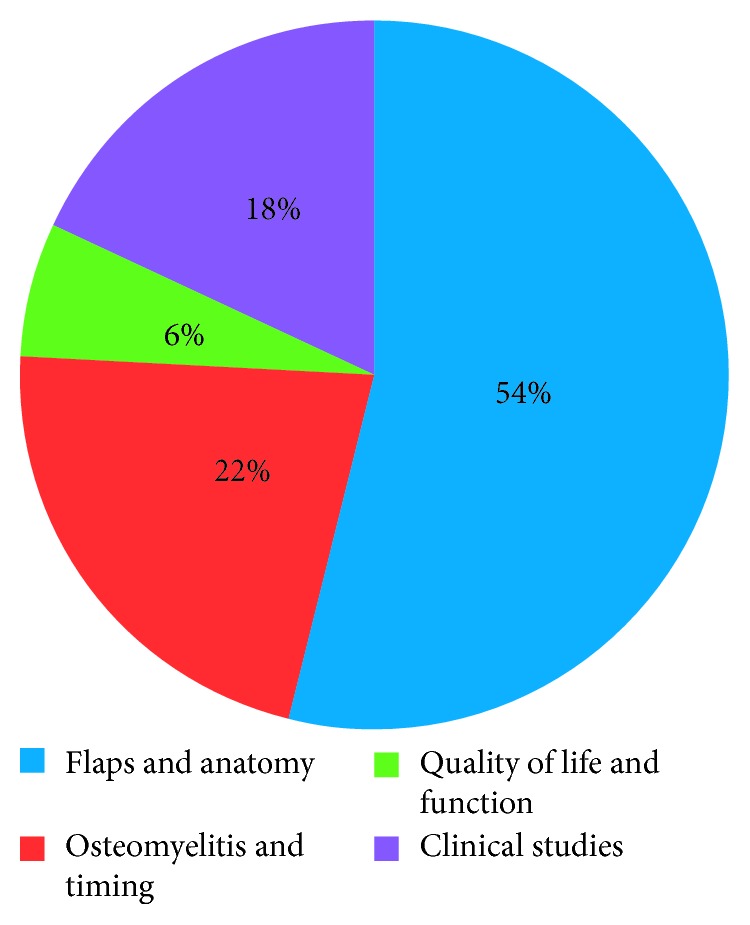
Categories of articles.

**Figure 2:**
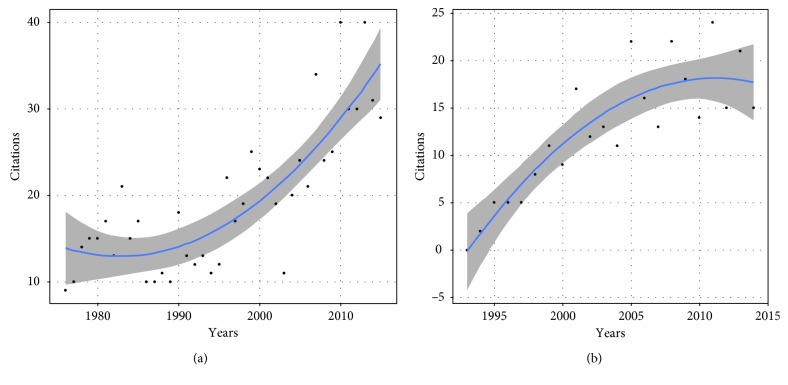
Example of citation patterns.

Articles were most often written by American medical centers (*n* = 22) (Table 3) and mostly published in PRS (*n* = 37); however, 17 countries were represented in 7 different peer-reviewed journals (Table 4). Of note, there was an article written by May et al. [25], and it is the only article published in a nonsurgical journal. All together, these “50 classics” have been cited 9599 times according to the WoS, 15536 times according to GS, and 9052 times in Scopus. The articles fall broadly into 4 different categories: anatomical studies and new flaps description; the timing for reconstruction and the prevention of osteomyelitis; the quality of life and functional results after reconstruction, and finally clinical evidence studies (Figure 1). The most recent articles were published in 2006 [26, 35, 45] and the oldest, 60 years earlier [52]. Within the 3 databases, the authors remain almost the same (Table 5). When using the mean citation number to sort the articles using the WoS database, the article written by Masquelet et al. [9] in 1992 is the most cited article, while the 2 oldest articles are at the bottom of the list (Table 6). Highly cited articles tend to remain highly cited, but the number of citations did not seem to be predictable (Figures 2(a) and 2(b)).

## 4. Discussion

The reconstruction and the coverage of wounds of the lower extremity is a tedious and challenging experience for surgeons involved. The bony support of the lower extremity, often prone to infection and malunion following trauma, has to be taken into consideration for its role in the gait biomechanics [12]. Furthermore, compared to the rest of the body, following loss of integument due to trauma, ulceration, or after skin resection, the lack of elasticity and poor vascularization of the surrounding skin associated with the lack of adipose tissue often prevents closure [19]. The reconstructive surgeon has to find a way to insure prompt coverage while replacing “like for like.” Multiple techniques have since been put into place to remedy this everlasting challenge.

In order to find the 50 most cited papers, we cross-referenced three search databases: the WoS, GS, and Scopus. These search tools were evaluated in an article written by Bakkalasi et al. in 2006 [60] by comparing databases, and findings indicated that they all returned unique materials and no search engine was superior over another. By cross-referencing, the articles were found in the different databases, and we were able to obtain a 100% concordance for the first 10 articles.

The articles found that, within the “most cited articles” list, all had a major impact in the evolution of reconstructive surgery of the extremity:

### 4.1. Muscle Flaps in the Treatment and Prevention of Osteomyelitis and the Timing of Surgery

The oldest article in this classic citation analysis dates back from 1946 and was written by Starks [52]. This is the first description of the use of a muscle flap for the treatment of chronic osteomyelitis. It is also a clear example of how authors do not always receive the recognition they deserve. The article by Mathes regarding the use of muscle flap in the treatment of infection [13], published 26 years later, is most often cited, and the use of this technique is often attributed to Ger [36] who also described the role of muscle flaps in the treatment and prevention of chronic osteomyelitis.

An important factor in the prevention of infection in lower extremity wounds is to find the appropriate timing. An important question is whether reconstruction should be early versus delayed and whether to reconstruct soft tissue and bone defect separately or not. Seven (7) studies investigating this issue appear in the 50 most cited articles for lower limb reconstructions. The use of muscle flap and free flap transfer not only gave reconstructive surgeons the possibility to carry these procedures in one step but also the possibility to close wounds that previously would have required an amputation. Taking into consideration Gustilo's classification [61] for open fractures, a new approach was then adopted. Byrd et al. [16, 21] were the pioneers to advocate the use of external osseous fixation and definitive early wound coverage (within the first 5 days of injury) for tibial shaft fractures. Yaremchuk et al. [22] corroborated these results in a long-term study. But the article written by Godina [10] had the most impact in this area. Though its findings have been disputed because of the comparison groups used and the associated injuries that could delay the intervention and therefore the statistical analysis, he is most often considered as the father of early wound coverage and the initiator of the collaboration between plastic surgeons and orthopaedic surgeons for the treatment of open fractures. Fischer et al. [40] in their article advocated that bone grafting should take place after complete soft tissue healing, but immediate intramedullary nailing had a lower risk of infection compared to delayed. Gopal et al. [18] went further by showing that immediate soft tissue reconstruction with internal bone fixation (<72 hours) was not only feasible but also caused less complications even for fractures with extensive tissue loss (grades IIIb and IIIc). More recently, reconstructions techniques have been modified by the arrival of new technologies such as the vacuum-assisted closure device (VAC) and skin substitutes such as Integra which has allowed closure of wounds without the need for the complex reconstructive procedure. DeFranzo et al. [15]. were the first to report a considerable cohort of patients treated with VAC therapy for closure of lower extremity wounds.

### 4.2. Anatomical Studies and New Flaps

As more flaps are being designed, the anatomical description behind these new surgical techniques is more and more precise. The oldest anatomical study for the lower extremity in this review was performed by Haertsch in 1981 [43] describing the cutaneous vessels of the legs. It was followed shortly after by the description of septocutaneous vessels by Carriquiry et al. in 1985 [29] and the introduction of the notion of angiosomes by Taylor and Pan [23, 62] that opened the doors to a multitude of new flap possibilities.

#### 4.2.1. Musculocutaneous Flaps

The article written in 1972 by Orticochea [39] describing a musculocutaneous flap allowed a way to have immediate wound coverage without the useful delay technique. It assured a good blood supply and a viable flap without the need for microvascular anastomoses. It also paved the way for the description of further musculocutaneous flap to be used for reconstruction. This was achieved with the gastrocnemius myocutaneous flap described by McCraw et al. [50] in 1976 and the medial gastrocnemius flap in 1978 by Feldman et al. [63]. These flaps were described as an attempt to find a local alternative for coverage of wounds of the lower thigh and below this level. It provided another option to the then over used cross leg flap and the growing field of microsurgery for free flap transfer. With its long arc of rotation, the medial gastrocnemius flap is still widely used for reconstruction of the knee area.

#### 4.2.2. Free Flap Transfer

2 articles had a major impact in this area for the reconstruction of lower extremity. Both Daniel and Taylor [11] and O'Brien et al. [20] described in 1973 the feasibility of the transfer of an island flap from a distant location for coverage of wounds in the lower extremity. Two years later in 1975, Taylor et al. described the first free vascularized bone graft for osseous reconstruction using a harvested fibula [8]. Buncke et al. went on in 1977 to transfer an island of skin with the underlying bone to reconstruct a tibia [48]. These 4 pioneer studies paved the way for further work concerning free bone transfer and osteoseptocutaneous flap (Wei et al. [64], Yoshimura et al. [33].)

#### 4.2.3. Fasciocutaneous Flap

The fasciocutaneous flap was introduced by Pontén in 1981 [12]. It gave reconstructive surgeons yet another mean for wound coverage in the lower extremity. It also gave the alternative to spare the muscle and have more versatility for wound coverage. Ponten showed that flaps could be vascularized through the fascia, allowing coverage from the knee down to the foot with the possibility to harvest larger flaps with no need for ratio consideration. Amarante et al. [28], Donski and Fogdestam [31], and Wee [44] are amongst the most cited authors for their contribution to the coverage of the distal lower limb wounds with their fasciocutaneous flap techniques.

#### 4.2.4. Neuroskin Island Flaps

Masquelet et al.'s article “Skin island flaps supplied by the vascular axis of the sensitive superficial nerves [9],” written in 1992, has the most citations per year. They introduced the concept of neuroskin flaps, and their findings allowed the description of many new flaps based on arteries accompanying the sural, saphenous, and peroneal nerves. It allowed sparing of the main underlying arteries while providing coverage for distal wounds such as with the distally based superficial sural artery flap [19]. Based on this study and previous anatomical description, Nakajima et al. then described flaps based on arteries accompanying the lesser saphenous vein [65].

#### 4.2.5. Soft Tissue Reconstruction of the Foot Area

The lack of soft tissue associated with the anatomy of the foot makes reconstruction of the foot even more challenging. Reconstruction can be obtained with a wide variety of free flaps or locoregional flaps, such as with the distally based superficial sural artery flap or the lateral supramalleolar flap which are in the list of most cited articles. The dorsalis arterialized flap described by McCraw in 1975 [32] is the only local flap for dorsal foot resurfacing that has been highly cited. The reason might be for its role in the reconstruction of heel defects, and the fact that it also allowed the description of the dorsalis pedis free flap which is a valuable neurovascular free flap in areas where thin skin is needed (i.e., hand reconstruction). Nevertheless, one limitation with this flap is the potential donor site complications.

### 4.3. Evidence-Based Medicine

A large proportion of highly cited articles are not actually new flaps description, but they bring the clinical evidence of these previously described flaps. It is critical for reconstructive surgeons to know the outcome of different techniques on a large scale of patients or to have comparison data of various techniques. These articles will most likely remain highly cited until larger series or more accurate ones are produced [17, 26, 33, 35, 41, 42, 47, 56].

### 4.4. Quality of Life and Functional Results after Reconstruction

Lower extremity reconstruction is not only complex because the osseous structure has to be properly aligned and covered with soft tissue. The goal of the reconstruction is also to restore the functionality. A few studies have looked specifically at the quality of life, the patient's satisfaction, and the functional results post-reconstruction. In their study, May et al. [27] were able to address the role of cutaneous sensibility in successful long-term reconstruction of weight bearing surfaces of the foot with a gait analysis, an issue that had not been previously solved. Francel et al. [38] in their classical article also looked at the functional outcome post-reconstruction but in relation to the quality of life of patients. This study helped reconstructive surgeons realize that a traumatic limb should not always be salvaged. More recently, studies from the LEAP group (lower extremity assessment project) have also produced data that are redefining reconstructive surgery by establishing evidence-based criteria on when to salvage a traumatic extremity. Although these studies bring insights into the field, they do not specifically address the subject set up in this article and were therefore not included.

These are not the only interesting studies that we were not able to include is this classical citation paper. Another highly referred article is the one written by Gustilo and Anderson [61], for the classification of open fractures. Although it is cited in a vast majority of papers dealing with reconstructive surgery of the lower limb, its topic is too diverse. Other articles simply did not have sufficient citations to be listed in this article but will probably be in a near future. Such is the case with propeller perforator flaps. They have gained approval in their use for lower limb reconstruction because of their low donor site morbidity [66].

Citation do not always give an author deserved recognition as many factors can cause an original article and an author to be incorrectly cited such as the language of publication and the access to the article [67–69]. Likewise, breakthrough techniques that become common knowledge stops being cited [70]. Also, some authors can be overcited for these same reasons or because of self-citations.

We did not evaluate all these multiple limitations inherent to such study. To counteract the possible time bias, we calculated the mean citation per year by including all years since the articles' first publication. Loonen et al. [71] defined the citable period as the first 16 years since the first publication and therefore had to exclude articles with a shorter citable period [72]. Joyce et al. adapted this 16 years citable period for more recent articles [6]. We noticed that, in the list of most cited articles for lower limb reconstructions, not only was the most recent article as recent as 2006, but some had a high citation rate only the first few years after their initial publication while a large number had their years with the most citation more than 16 years after the initial publication date. When plotting the number of citations according to the years, there is no clear correlation and the citation numbers were different than expected. Furthermore, the years when an article is most cited cannot be retrospectively explained. Various studies have designed models to predict citation patterns [73, 74], but predicting citation numbers still remain challenging.

Most highly cited articles were published in PRS which is currently the plastic surgery journal with the highest impact factor. Even though authors aim for a high impact factor journal when submitting an article, they have to keep in mind that it is a biased number. A more reliable way to identify the impact of an article is by observing how it is cited over time by other authors. With effective search engines such as Medline and Google Scholar and the possibility of open access publications, if an article brings an important contribution to its field, it will become highly cited over time. Authors should not focus on being published in prestigious journals but rather evaluate their contribution by their number of citations, which is now mentioned in platforms such as ResearchGate.

## 5. Conclusion

Flap designs and reconstructive techniques of the lower limb have been influenced by a wide variety of articles. Most highly cited articles have been anatomical studies allowing the design of new flaps. Only a small portion of articles are focusing on the effect of these procedures on the quality of life of patients. Furthermore, the timing of these procedures has also been taken into consideration. Clinical studies have then been put in place to validate these results. Clinician and researchers alike need also to focus on this neglected area of research and follow the trend started by our predecessors towards a minimally invasive and safe surgery with the best functional results for our patients without focusing on the journal of publication.

## Figures and Tables

**Table 1:** Ranking according to WoS, GS, Scopus, and years with the most citations.

Rank		Author and title	Year	Top year	CN WoS	CN GS	CN Scopus	MC WoS
1		Taylor et al. The free vascularized bone graft: a clinical extension of microvascular techniques [8].	1975	2013	829	1263	760	20.72
2		Masquelet et al. Skin island flaps supplied by the vascular axis of the sensitive superficial nerves: anatomic study and clinical experience in the leg [9].	1992	2011	530	1448	411	23.04
3		Godina. Early microsurgical reconstruction of complex trauma of the extremities [10].	1986	2007	553	1016	575	19.06
4		Daniel and Taylor. Distant transfer of an island flap by microvascular anastomoses: a clinical technique [11].	1973	1979	438	652	352	8.42
5		Pontén. The fasciocutaneous flap: its use in soft tissue defects of the lower leg [12].	1981	1990	433	729	447	12.73
6		Mathes. Use of the muscle flap in chronic osteomyelitis: experimental and clinical correlation [13].	1982	1996	348	451	314	10.54
7		Wei et al. Fibular osteoseptocutaneous flap: anatomic study and clinical application [14].	1986	2010	256	345	276	8.83
8		DeFranzo et al. The use of vacuum-assisted closure therapy for the treatment of lower-extremity wounds with exposed bone [15].	2001	2006	249	506	272	17.78
9		Byrd et al. Management of open tibial fractures [16].	1985	1999, 2008	239	390	225	7.97
10		Khouri and Shaw. Reconstruction of the lower extremity with microvascular free flaps: a 10-year experience with 304 consecutive cases [17].	1989	1995	229	289	223	8.81
11		Gopal et al. Fix and flap: the radical orthopaedic and plastic treatment of severe open fractures of the tibia [18].	2000	2014	199	380	227	13.27
12		Hasegawa et al. The distally based superficial sural artery flap [19].	1994	2008	196	378	229	9.33
13		O'Brien et al. Successful transfer of a large island flap from the groin to the foot by microvascular anastomoses [20].	1973	1976	180	222	141	4.28
14		Byrd et al. The management of open tibial fractures with associated soft-tissue loss: external pin fixation with early flap coverage [21].	1981	1997	172	256	166	5.06
15		Yaremchuk et al. Acute and definitive management of traumatic osteocutaneous defects of the lower extremity [22].	1987	2010	166	256	169	5.93
16		Taylor and Pan. Angiosomes of the leg: anatomic study and clinical implications [23].	1998	2013	163	298	122	9.59
17		Taylor and Watson. One-stage repair of compound leg defects with free, revascularized flaps of groin skin and iliac bone [24].	1982	1989	161	175	119	4.88
18		May et al. Microvascular transfer of free tissue for closure of bone wounds of the distal lower extremity [25].	1982	1989	161	175	119	4.88
19		Yazar et al. Outcome comparison between free muscle and free fasciocutaneous flaps for reconstruction of distal third and ankle traumatic open tibial fractures [26].	2006	2009, 2011	156	95	99	17.33
20		May et al. Free microvascular muscle flaps with skin graft reconstruction of extensive defects of the foot—a clinical and gait analysis study [27].	1985	1986	152	227	151	5.07
21		Amarante et al. A new distally based fasciocutaneous flap of the leg [28].	1986	1990	151	200	157	5.20
22		Carriquiry et al. An anatomic study of the septocutaneous vessels of the leg [29].	1985	1990	150	218	144	5
23		Yazar et al. One-stage reconstruction of composite bone and soft-tissue defects in traumatic lower extremities [30].	2004	2010	149	216	140	13.54
24		Donski and Fogdestam. Distally based fasciocutaneous flap from the sural region: a preliminary report [31].	1983	1990	148	242	168	4.62
25		McCraw et al. The dorsalis pedis arterialized flap: a clinical study [32].	1975	1979	146	258	140	3.65
26		Yoshimura et al. Peroneal flap for reconstruction in the extremity [33].	1984	1990	140	176	142	4.52
27		Georgiadis et al. Open tibial fractures with severe soft-tissue loss-limb salvage compared with below-the-knee amputation [34]	1993	1997	140	228	140	6.36
28		Parrett et al. Lower extremity trauma: trends in the management of soft-tissue reconstruction of open tibia-fibula fractures [35]	2006	2011, 2015	139	240	139	15.44
29		Ger. Muscle transposition for treatment and prevention of chronic posttraumatic osteomyelitis of the tibia [36].	1977	1983	134	172	93	3.53
30		Masquelet et al. The lateral supramalleolar flap [37].	1988	2001	134	246	145	4.96
31		Francel et al. Microvascular soft-tissue transplantation for reconstruction of acute open tibial fractures: timing of coverage and long-term functional results [38].	1992	2014	134	204	143	5.83
32		Orticochea. The musculo-cutaneous flap method: an immediate and heroic substitute for the method of delay [39].	1972	1983	133	178	97	3.09
33		Fischer et al. The timing of flap coverage, bone-grafting, and intramedullary nailing in patients who have a fracture of the tibial shaft with extensive soft-tissue injury [40].	1991	2012	132	271	152	5.5
34		Baumeister et al. A realistic complication analysis of 70 sural artery flaps in a multimorbid patient group [41].	2003	2008	128	251	153	10.67
35		Serafin et al. Comparison of free flaps with pedicled flaps for coverage of defects of the leg or foot [42].	1977	1979	126	174	114	3.31
36		Haertsch. The blood supply to the skin of the leg: a post-mortem investigation [43].	1981	1986	125	216	109	3.68
37		Wee. Reconstruction of the lower leg and foot with the reverse-pedicled anterior tibial flap: preliminary report of a new fasciocutaneous flap [44].	1986	1990	123	163	116	4.24
38		Attinger et al. Angiosomes of the foot and ankle and clinical implications for limb salvage: reconstruction, incisions and revascularization [45]	2006	2013	123	272	162	13.67
39		Anthony et al. The muscle flap in the treatment of chronic lower extremity osteomyelitis: results in patients over 5 years after treatment [46]	1991	2004	122	173	125	5.08
40		Serafin et al. Reconstruction of the lower extremity with vascularized composite tissue: improved tissue survival and specific indications [47].	1980	1989	121	146	100	3.46
41		Buncke et al. Free osteocutaneous flap from a rib to the tibia [48].	1977	1982	121	166	70	3.18
42		Nakajima et al. Accompanying arteries of the lesser saphenous vein and sural nerve: anatomic study and its clinical applications [49].	1999	2011	119	230	129	7.0
43		McCraw et al. Versatile gastrocnemius myocutaneous flap [50]	1978	1984	115	168	91	3.11
44		Cavadas et al. The medial sural artery perforator free flap [51]	2001	2007	108	173	57	7.2
45		Starks The use of pedicled muscle flaps in the surgical treatment of chronic osteomyelitis resulting from compound fractures [52].	1946	1993	108	126	75	1.54
46		Jeng and Wei. Distally based sural island flap for foot and ankle reconstruction [53].	1997	2008	107	185	131	5.94
47		Morrison et al. The instep of the foot as a Fascicutaneous island and as a free flap for heel defects [54].	1983	1986	106	157	104	4.72
48		Almeida et al. Reverse-flow island sural flap [55].	2002	2007, 2008	104	186	114	8
49		Heller and Levin. Lower extremity microsurgical reconstruction [56].	2001	2009	102	169	65	7.28
50		Yilmaz et al. The distally based superficial sural artery island flap: clinical experiences and modifications [57].	1998	2006	101	181	140	5.94

Web of Science (Wos); Google Scholar (GS); citation number (CN); mean citation (MC); top year: year with the most citations.

**Table 2:** Authors with multiple articles as the first author.

Authors	Number of articles
Taylor	3
McCraw	2
Masquelet	2
Mathes	2
May	2
Serafin	2
Yazar	2
Total	15

**Table 3:** List of countries of publication.

Countries	Number of articles
USA	22
Australia	6
Japan	3
China	1
Taiwan	4
France	2
England	2
Switzerland	1
Germany	1
Colombia	1
Sweden	1
Turkey	1
Portugal	1
Yugoslavia	1
Spain	1
Canada	1
Brazil	1
Total	50

**Table 4:** List of journals.

Journal name	Number
Plastic and Reconstructive Surgery	37
Journal of Plastic Reconstructive and Aesthetic Surgery	5
American Journal of Bone and Joint Surgery	4
British Journal of Bone and Joint surgery	1
Scandinavian Journal of Plastic Surgery	1
Journal of Trauma Injury Infection and Critical care	1
The New England Journal of Medicine	1
Total	50

**Table 5:** Comparison of the 10 most cited articles ranked with different search engines (WoS, GS, and Scopus).

Rank	WoS	GS	Scopus
1	Taylor et al. The free vascularized bone graft: a clinical extension of microvascular techniques [8].	Masquelet et al. Skin island flaps supplied by the vascular axis of the sensitive superficial nerves: anatomic study and clinical experience in the leg [9].	Taylor et al. The free vascularized bone graft: a clinical extension of microvascular techniques [8].
2	Masquelet et al. Skin island flaps supplied by the vascular axis of the sensitive superficial nerves: anatomic study and clinical experience in the leg [9].	Taylor et al. The free vascularized bone graft: a clinical extension of microvascular.	Godina. Early microsurgical reconstruction of complex trauma of the extremities [10].
3	Godina Early microsurgical reconstruction of complex trauma of the extremities [10].	Godina. Early microsurgical reconstruction of complex trauma of the extremities [10].	Masquelet et al. Skin island flaps supplied by the vascular axis of the sensitive superficial nerves: anatomic study and clinical experience in the leg.
4	Daniel and Taylor. Distant transfer of an island flap by microvascular anastomoses: a clinical technique [11].	Pontén. The fasciocutaneous flap: its use in soft tissue defects of the lower leg [12].	Pontén. The fasciocutaneous flap: its use in soft tissue defects of the lower leg [12].
5	Pontén. The fasciocutaneous flap: its use in soft tissue defects of the lower leg [12].	Daniel and Taylor. Distant transfer of an island flap by microvascular anastomoses: a clinical technique [11].	Daniel and Taylor. Distant transfer of an island flap by microvascular anastomoses: a clinical technique [11].
6	Mathes. Use of the muscle flap in chronic osteomyelitis: experimental and clinical correlation [13].	DeFranzo et al. The use of vacuum-assisted closure therapy for the treatment of lower-extremity wounds with exposed bone [15].	Mathes. Use of the muscle flap in chronic osteomyelitis: experimental and clinical correlation [13].
7	Wei et al. Fibular osteoseptocutaneous flap: anatomic study and clinical application [14].	Mathes. Use of the muscle flap in chronic osteomyelitis: experimental and clinical correlation [13].	Wei et al. Fibular osteoseptocutaneous flap: anatomic study and clinical application [14].
8	DeFranzo et al. The use of vacuum-assisted closure therapy for the treatment of lower-extremity wounds with exposed bone [15].	Wei et al.. Fibular osteoseptocutaneous flap: anatomic study and clinical application [14].	DeFranzo et al. The use of vacuum-assisted closure therapy for the treatment of lower-extremity wounds with exposed bone [15].
9	Byrd et al. Management of open tibial fractures [16].	Byrd et al. Management of open tibial fractures [16].	Byrd et al. Management of open tibial fractures [16].
10	Khouri and Shaw. Reconstruction of the lower extremity with microvascular free flaps: a 10-year experience with 304 consecutive cases [17].	Khouri and Shaw. Reconstruction of the lower extremity with microvascular free flaps: a 10-year experience with 304 consecutive cases [17].	Khouri and Shaw. Reconstruction of the lower extremity with microvascular free flaps: a 10-year experience with 304 consecutive cases [17].

Web of Science (Wos); Google Scholar (GS).

**Table 6:** Ranking according to the mean citation number.

Rank	Author and title	Mean citation
1	Masquelet et al. Skin island flaps supplied by the vascular axis of the sensitive superficial nerves: anatomic study and clinical experience in the leg [9].	23.04
2	Taylor et al. The free vascularized bone graft: a clinical extension of microvascular [8].	20.72
3	Godina. Early microsurgical reconstruction of complex trauma of the extremities [10].	19.06
4	DeFranzo et al. The use of vacuum-assisted closure therapy for the treatment of lower-extremity wounds with exposed bone [15].	17.78
5	Yazar et al. Outcome comparison between free muscle and free fasciocutaneous flaps for reconstruction of distal third and ankle traumatic open tibial fractures [26].	17.33
6	Parrett et al. Lower extremity trauma: trends in the management of soft-tissue reconstruction of open tibia-fibula fractures [35].	15.44
7	Attinger et al. Angiosomes of the foot and ankle and clinical implications [45].	13.67
8	Yazar et al. One-stage reconstruction of composite bone and soft-tissue defects in traumatic lower extremities [30].	13.54
9	Gopal et al. Fix and flap: the radical orthopaedic and plastic treatment of severe open fractures of the tibia [18].	13.27
10	Pontén. The fasciocutaneous flap: its use in soft tissue defects of the lower leg [12].	12.73
11	Baumeister et al. A realistic complication analysis of 70 sural artery flaps in a multimorbid patient group [41].	10.67
12	Mathes et al. Use of the muscle flap in chronic osteomyelitis: experimental and clinical correlation [58].	10.54
13	Taylor et al. Angiosomes of the leg: anatomic study and clinical implications [23].	9.59
14	Hasegawa et al. The distally based superficial sural artery flap [19].	9.33
15	Wei et al. Fibula osteoseptocutaneous flap for reconstruction of composite mandibular defects [14].	8.83
16	Khouri and Shaw. Reconstruction of the lower extremity with microvascular free flaps: a 10-year experience with 304 consecutive cases [17].	8.81
17	Daniel and Taylor. Distant transfer of an island flap by microvascular anastomoses: a clinical technique [11].	8.42
18	Almeida et al. Reverse-flow island sural flap [55].	8.0
19	Byrd et al. Management of open tibial fractures [16].	7.97
20	Heller and Levin Lower extremity microsurgical reconstruction [56].	7.28
21	Cavadas et al. The medial sural artery perforator free flap [51].	7.2
22	Nakajima et al. Accompanying arteries of the lesser saphenous vein and sural nerve: anatomic study and its clinical applications [49].	7.0
23	Georgiadis et al. Open tibial fractures with severe soft-tissue loss-limb salvage compared with below-the-knee amputation [34].	6.36
24	Jeng and Wei. Distally based sural island flap for foot and ankle reconstruction [53].	5.94
25	Yilmaz et al. The distally based superficial sural artery island flap: clinical experiences and modifications [57].	5.94
26	Yaremchuk et al. Acute and definitive management of traumatic osteocutaneous defects of the lower extremity [22].	5.93
27	Francel et al. Microvascular soft-tissue transplantation for reconstruction of acute open tibial fractures: timing of coverage and long-term functional results [38].	5.83
28	Fischer et al. The timing of flap coverage, bone-grafting, and intramedullary nailing in patients who have a fracture of the tibial shaft with extensive soft-tissue injury [40].	5.5
29	Amarante et al. A new distally based fasciocutaneous flap of the leg [28].	5.20
30	Anthony et al. The muscle flap in the treatment of chronic lower extremity osteomyelitis: results in patients over 5 years after treatment [46].	5.08
31	May et al. Free microvascular muscle flaps with skin graft reconstruction of extensive defects of the foot—a clinical and gait analysis study [27].	5.07
32	Byrd et al. The management of open tibial fractures with associated soft-tissue loss: external pin fixation with early flap coverage [21].	5.06
33	Carriquiry et al. An anatomic study of the septocutaneous vessels [29].	5.0
34	Masquelet et al. The lateral supramalleolar flap [37].	4.96
35	May et al. Microvascular transfer of free tissue for closure of bone wounds of the distal lower extremity [25].	4.88
36	Taylor and Pan. Angiosomes of the leg: anatomic study and clinical implications [23].	4.88
37	Morrison et al. The instep of the foot as a fasciocutaneous island and as a free flap for heel defects [59].	4.72
38	Donski and Fogdestam. Distally based fasciocutaneous flap from the sural region: a preliminary report [31].	4.62
39	Yoshimura et al. Peroneal flap for reconstruction in the extremity [33].	4.52
40	O'Brien et al. Successful transfer of a large island flap from the groin to the foot by microvascular anastomoses [20].	4.28
41	Wee. Reconstruction of the lower leg and foot with the reverse-pedicled anterior tibial flap: preliminary report of a new fasciocutaneous flap [44].	4.24
42	Haertsch. The blood supply to the skin of the leg: a post-mortem [43].	3.68
43	McCraw et al. The dorsalis pedis arterialized flap: a clinical study [32].	3.65
44	Ger. Muscle transposition for treatment and prevention of chronic posttraumatic osteomyelitis of the tibia [36].	3.53
45	Serafin et al. Reconstruction of the lower extremity with vascularized composite tissue: improved tissue survival and specific indications [47].	3.46
46	Serafin et al. Comparison of free flaps with pedicled flaps for coverage of defects of the leg or foot [42].	3.31
47	Buncke et al. Free osteocutaneous flap from a rib to the tibia [48].	3.18
48	McCraw et al. Versatile gastrocnemius myocutaneous flap [50].	3.11
49	Orticochea. The musculo-cutaneous flap method: an immediate and heroic substitute for the method of delay [39].	3.09
50	Starks. The use of pedicled muscle flaps in the surgical treatment of chronic osteomyelitis resulting from compound fractures [52].	1.54
